# Network Pharmacology and Bioinformatics Study of Six Medicinal Food Homologous Plants Against Colorectal Cancer

**DOI:** 10.3390/ijms26030930

**Published:** 2025-01-23

**Authors:** Xinyue Zhao, Jian Xiu, Hengzheng Yang, Weiwei Han, Yue Jin

**Affiliations:** 1 Key Laboratory for Molecular Enzymology and Engineering of Ministry of Education, School of Life Science, Jilin University, Changchun 130012, China; zhaoxinyue20@mails.jlu.edu.cn (X.Z.); xiujian1321@mails.jlu.edu.cn (J.X.); yanghz24@mails.jlu.edu.cn (H.Y.); 2 Edmond H. Fischer Signal Transduction Laboratory, School of Life Sciences, Jilin University, Changchun 130012, China; 3 National Engineering Laboratory of AIDS Vaccine, School of Life Sciences, Jilin University, Changchun 130012, China

**Keywords:** network pharmacology, bioinformatics, traditional Chinese medicine, medicinal food homologous plants, colorectal cancer

## Abstract

Integrating network pharmacological analysis and bioinformatic techniques, this study systematically investigated the molecular mechanisms of six medicinal food homologous plants (*Astragalus membranaceus*, *Ganoderma lucidum*, *Dioscorea opposite*, *Curcuma longa*, *Glycyrrhiza uralensis*, and *Pueraria lobata*) against colorectal cancer. Through screening the TCMSP database, 303 active compounds and 453 drug targets were identified. By integrating differential expression gene analysis with WGCNA on the GSE41258 dataset from the GEO database, 49 potential therapeutic targets were identified. GO and KEGG enrichment analyses demonstrated that these targets are primarily involved in drug response, fatty acid metabolism, and key cancer-related pathways. Cross-validation using three machine learning algorithms—LASSO regression, SVM-RFE, and Random Forest—pinpointed four critical target genes: CA1, CCND1, CXCL2, and EIF6. Further, CIBERSORT immune infiltration analysis revealed strong associations between these core genes and the tumor immune microenvironment in colorectal cancer patients, notably in modulating M0 macrophage infiltration and mast cell activity. Molecular docking analyses confirmed robust binding interactions between active compounds and core target proteins. This study systematically elucidated the molecular mechanisms of six medicinal food homologous plants against colorectal cancer, providing scientific evidence for their rational clinical application.

## 1. Introduction

Colorectal cancer (CRC) remains a major global health threat, with rising incidence and mortality rates. As reported by GLOBOCAN 2023, CRC ranks third in global cancer incidence and second in cancer-related mortality [1,2]. The disease burden is particularly pronounced in developed countries, while rapidly increasing trends are observed in many developing nations due to the adoption of Western lifestyles and dietary habits. Despite the continuous development and improvement of standardized treatment protocols including surgical resection, chemoradiotherapy, and molecular targeted therapy, clinical outcomes for CRC patients remain suboptimal [3,4,5]. The five-year survival rate for metastatic CRC remains below 15%, primarily due to factors such as tumor heterogeneity, drug resistance, severe side effects, and high treatment costs [6,7,8]. Furthermore, conventional treatments often lead to decreased quality of life and substantial economic burden for patients [9]. Therefore, developing new therapeutic drugs with proven efficacy, good safety profiles, and favorable accessibility holds significant clinical translational value.

With thousands of years of practice, traditional Chinese medicine (TCM) has developed extensive expertise in cancer prevention and therapy. Modern clinical research increasingly highlights its distinct advantages in treating diverse tumors [10,11]. A randomized controlled trial on non-small cell lung cancer demonstrated that integrating the Chinese herbal compound Shenlingcao with standard chemotherapy significantly improved patients’ quality of life and alleviated cancer-related symptoms [12,13]. In hepatocellular carcinoma, the latest clinical evidence similarly supports the positive role of Chinese medicine in reducing incidence and improving prognosis [14]. Specifically in CRC treatment, several meta-analyses have demonstrated that TCM, when used as adjuvant therapy, can effectively reduce chemotherapy-related adverse effects, enhance treatment efficacy, and improve patient survival rates. These studies provide important evidence-based medical support for the application of TCM in comprehensive cancer treatment.

Medicinal food homologous plants represent a distinctive therapeutic resource in the TCM treasury, featuring the notable advantage of dual use as both medicine and food, with deep historical foundations in Chinese traditional medical practice [15]. The six medicinal food homologous plants focused on in this study (*Astragalus membranaceus*, *Ganoderma lucidum*, *Dioscorea opposite*, *Curcuma longa*, *Glycyrrhiza uralensis*, and *Pueraria lobata*) have all demonstrated significant anti-tumor activity in experimental research. Among them, astragalus polysaccharides exert anti-tumor effects through immune system regulation, particularly by enhancing natural killer cell activity and promoting T lymphocyte proliferation [16]. *Ganoderma polysaccharides* improve immune function and suppress tumor growth by inducing cell cycle arrest and promoting apoptosis through various mechanisms [17,18]. By influencing the gut microecological system and inflammatory pathways, *Dioscorea opposite* exhibits anti-tumor properties. Curcumin, derived from *Curcuma longa*, drives tumor cell apoptosis through mechanisms involving NF-κB, STAT3, and PI3K/Akt signaling [19,20,21]. The bioactive components in *Glycyrrhiza uralensis*, such as glycyrrhizin and licochalcone A, demonstrate promising anti-tumor potential through anti-inflammatory and anti-angiogenic mechanisms [22,23]. The isoflavones in *Pueraria lobata* exhibit significant anti-proliferative activity and promote apoptosis across various cancer cell lines [24,25,26]. However, the molecular mechanisms underlying the synergistic effects of these herbs in combating colorectal cancer remain inadequately investigated.

Network pharmacology, as an integrative systems biology research method, effectively integrates with the fundamental principles of TCM, which operates through multiple components and diverse biological targets, providing an innovative research strategy for elucidating the mechanisms of complex drug systems [27,28,29,30]. This approach marks a significant shift from the conventional “one drug, one target” model to a holistic “network target, multi-component” strategy, aligning more closely with the intricate nature of disease mechanisms and therapeutic actions. Advances in bioinformatics, especially the integration of machine learning algorithms in drug discovery, now provide robust tools to unravel the complex action networks of traditional Chinese medicine. Advanced computational methods such as molecular docking, network analysis, and artificial intelligence algorithms enable the prediction and validation of drug–target interactions at an unprecedented scale. This interdisciplinary research approach enables us to understand the complex interaction networks of drug-target-pathway-disease at a systemic level.

This study applies network pharmacology and bioinformatics methods to explore the molecular mechanisms of six medicinal food homologous plants in colorectal cancer therapy. Active components and targets were identified from the Traditional Chinese Medicine Systems Pharmacology Database and Analysis Platform (TCMSP) [31] and integrated with gene expression profiles from the Gene Expression Omnibus (GEO) database [32]. Comprehensive analyses, including protein–protein interaction networks, pathway enrichment, machine learning techniques, and immune infiltration evaluations, revealed the synergistic effects of these herbs. Advanced methods like Least Absolute Shrinkage and Selection Operator (LASSO) regression [33], Support Vector Machine-Recursive Feature Elimination (SVM-RFE) [34], and Random Forest (RF) [35] were used to pinpoint robust therapeutic targets and biomarkers.

**Figure 1:**
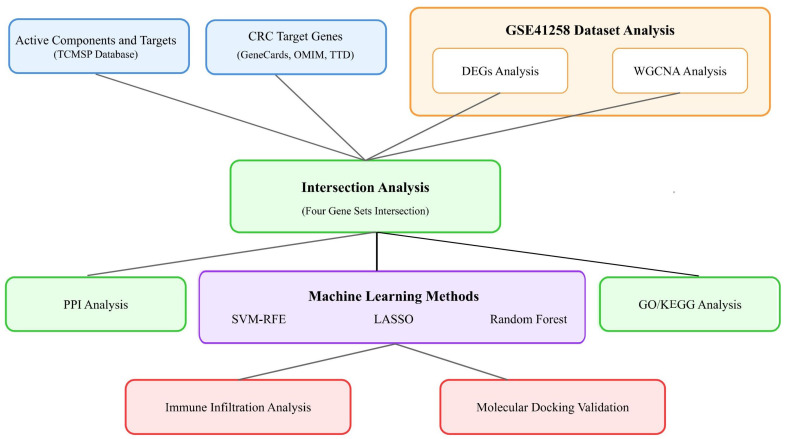
Overview of experimental design and analysis pipeline.

Furthermore, immune infiltration analysis provides novel insights into the immunomodulatory effects of these herbs in the tumor microenvironment. This research not only helps elucidate the molecular mechanisms of medicinal food homologous plants against colorectal cancer but also provides scientific evidence for their rational clinical application, while offering new research perspectives and methodological references for multi-target drug development based on network pharmacology. This study’s findings may contribute to personalized CRC treatments, improving both clinical outcomes and patient quality of life. Figure 1 presents a schematic overview of our methodological workflow.

## 2. Results

### 2.1. Screening of Bioactive Compounds and Therapeutic Targets

**Figure 2:**
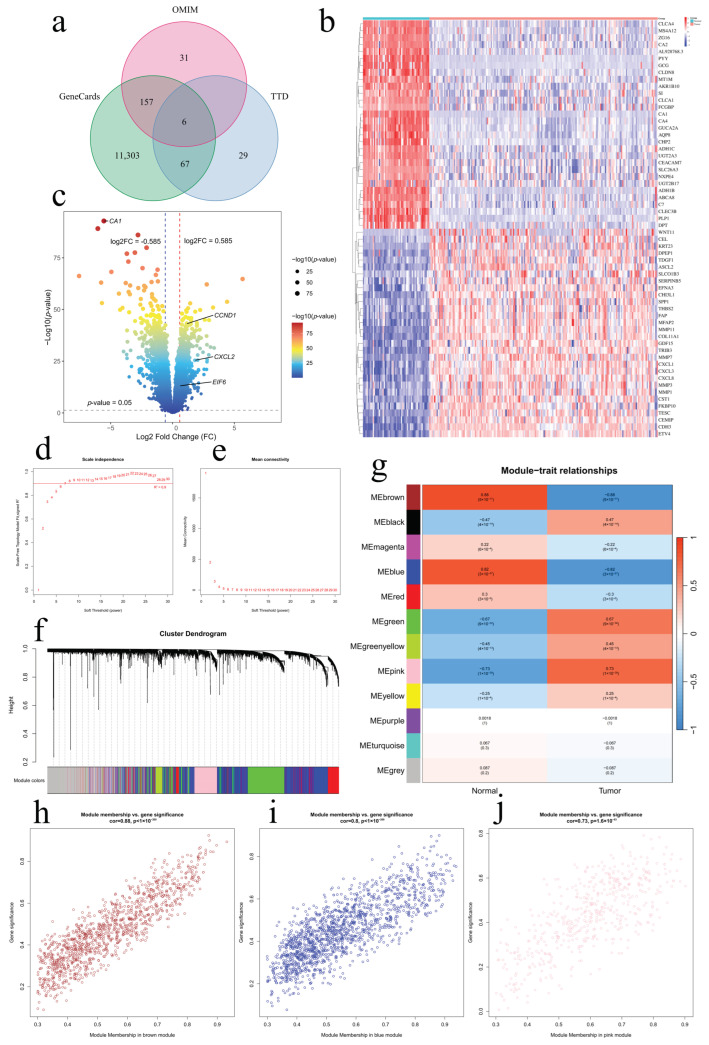
Identification of CRC-related targets. (**a**) Venn diagram of database-derived targets. (**b**) Heatmap of the 60 most significant DEGs. (**c**) Volcano plot. (**d**) Scale-free topology analysis in WGCNA. (**e**) Mean connectivity analysis in WGCNA. (**f**) Clustering dendrogram of WGCNA genes and module differentiation. Different modules are represented by distinct colors. (**g**) Module–trait relationship analysis diagram of 12 modules. Correlation plots depicting GS–MM relationships for CRC in brown (**h**), blue (**i**), and pink (**j**) modules.

**Figure 3:**
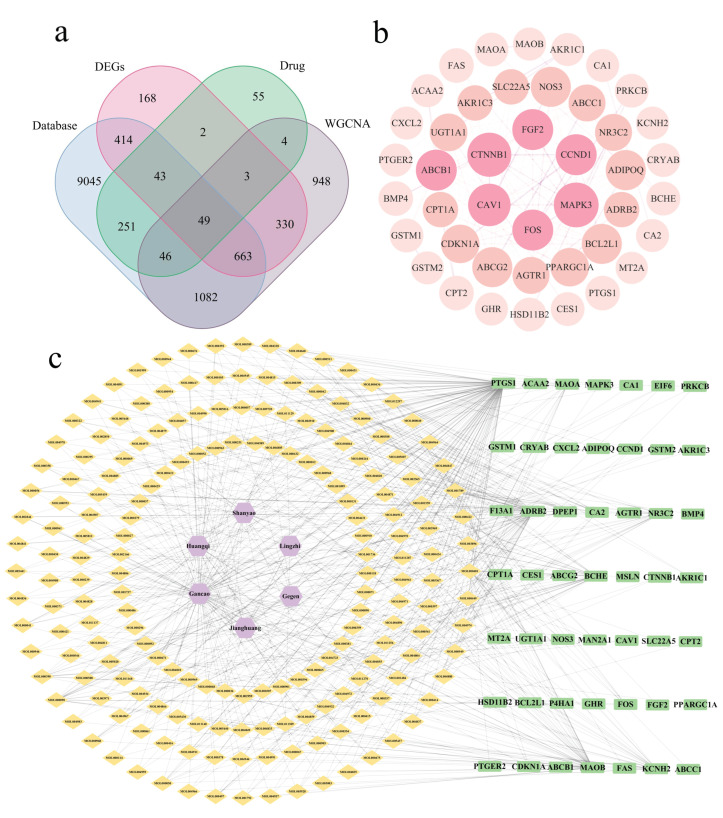
Computational analysis of therapeutic targets and interaction networks in CRC. (**a**) Venn analysis for target identification. (**b**) Visualization of cross-target PPI network. (**c**) Drug–CRC network diagram. Purple pentagons represent drug categories. Orange diamonds represent active components. Green squares represent intersection genes. Connecting lines indicate associations between nodes.

Using the TCMSP database, 303 active components and 453 drug targets were identified from six medicinal food homologous plants (Supplementary Tables S1 and S2). A total of 11,593 CRC target genes were obtained from public databases such as GeneCards, OMIM, and TTD (Figure 2a). Differential expression analysis of the GSE41258 dataset identified 1672 differentially expressed genes, which we visualized using a heatmap and volcano plots (Figure 2b,c; Supplementary Table S2). Using the GSE41258 dataset, gene co-expression networks were constructed via Weighted Gene Co-expression Network Analysis (WGCNA) to identify CRC-related key gene modules. The soft threshold was determined to be 7 (Figure 2d,e) through the evaluation of scale independence and mean connectivity patterns. The gene co-expression network analysis incorporated hierarchical clustering and dynamic tree-cutting algorithms to identify distinct modules. The integration of similar modules yielded 12 unique gene clusters, visualized through a dendrogram (Figure 2f). Among these, the brown, blue, and pink modules demonstrated robust correlations with CRC/non-CRC phenotypes (Figure 2g). Statistical evaluation revealed strong positive associations between Gene Significance (GS) and Module Membership (MM) metrics across CRC-related genes in these three modules (Figure 2h–j). The genes from the three modules were merged to obtain WGCNA results (Supplementary Table S2). Through intersection calculation, 49 potential therapeutic targets were identified (Figure 3a). These results suggest that these six herbs may act synergistically through multiple active components on multiple targets.

### 2.2. Establishment of Protein–Protein Interaction and Drug–Disease Networks

A PPI network comprising the 49 intersection genes was constructed using the STRING database and subsequently visualized through Cytoscape (Figure 3b). Additionally, we constructed a drug–colorectal cancer network incorporating herb types, their active components, and the 49 overlapping target genes (Figure 3c).

### 2.3. Gene Ontology and KEGG Pathway Analysis

**Figure 4:**
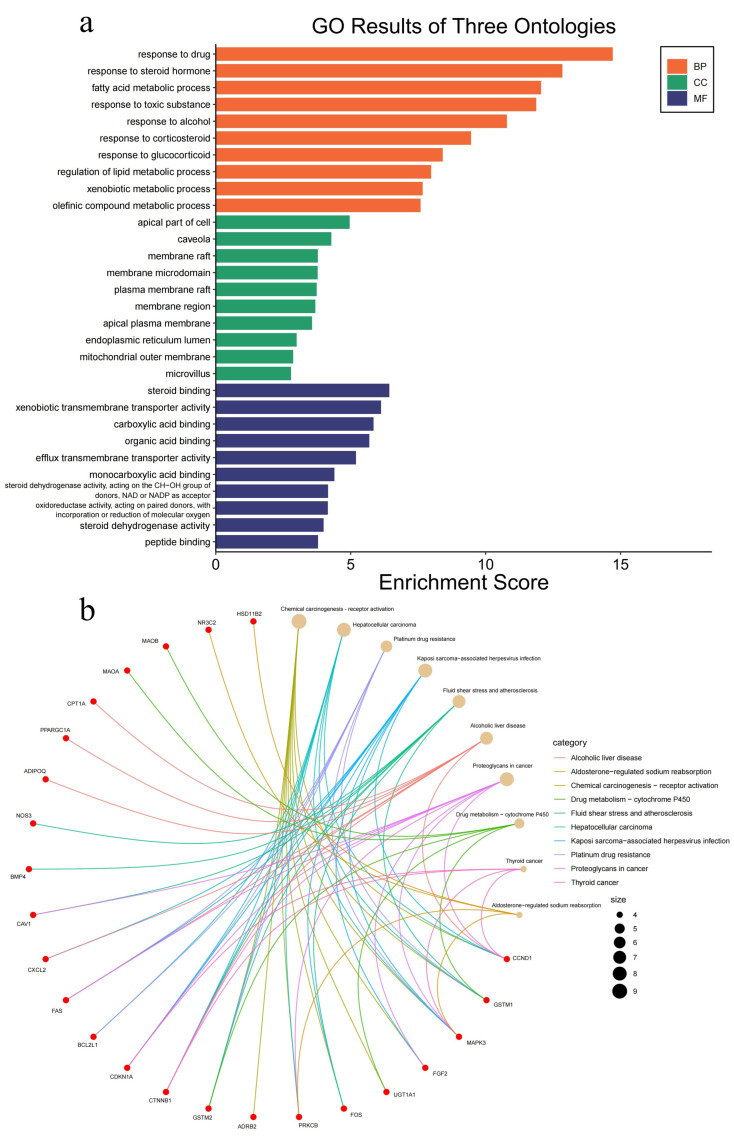
Functional enrichment analysis of six traditional Chinese herbs in colorectal cancer. (**a**) Bar plot from GO enrichment analysis. (**b**) Cnet plot from KEGG pathway enrichment analysis.

Gene ontology (GO) enrichment analysis (Figure 4a) demonstrated that the target genes were predominantly linked to biological processes (BP), including drug response, fatty acid metabolism, alcohol response, and hormone response. Cellular component (CC) categories included the apical part of cells, caveolae, membrane rafts, endoplasmic reticulum lumen, outer mitochondrial membrane, and microvilli. Molecular function (MF) terms highlighted steroid binding, xenobiotic transmembrane transport, monocarboxylic acid binding, and various dehydrogenase activities. Kyoto Encyclopedia of Genes and Genomes (KEGG) pathway enrichment analysis (Figure 4b) revealed significant involvement in cancer-related pathways, including colorectal cancer and other oncogenic pathways, consistent with the established mechanisms of CRC pathogenesis.

### 2.4. Identification and Validation of Core Genes

**Figure 5:**
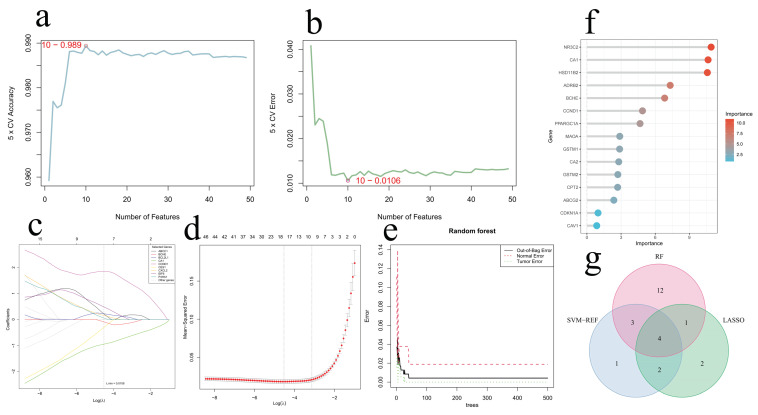
Hub gene identification through machine learning approaches. (**a**) The accuracy rate curves derived from five-fold cross-validation using the SVM-RFE algorithm. (**b**) The error rate curves obtained from five-fold cross-validation with the SVM-RFE algorithm. (**c**) The coefficient diagrams produced by LASSO analysis. (**d**) The regularization diagrams generated through LASSO analysis. (**e**) The error rate curves from RF method. (**f**) The importance evaluation from RF method. (**g**) Intersection analysis of essential genes discovered via multiple ML algorithms.

Using three machine learning methods, we screened 49 intersecting genes to assess their ability to differentiate CRC samples from controls in the GSE41258 dataset, ultimately identifying hub genes. The SVM-RFE algorithm identified a ten-gene signature, comprising CA1, CA2, NR3C2, CES1, CCND1, CXCL2, BCL2L1, HSD11B2, EIF6, and ADIPOQ (Figure 5a,b). Through LASSO analysis, 9 genes (ABCC1, BCHE, BCL2L1, CA1, CCND1, CES1, CXCL2, EIF6, and P4HA1) were filtered as core target genes (Figure 5c,d). Based on the RF algorithm, and calculating variable importance values for potential target genes with a threshold of 0.5, we obtained 20 core genes (NR3C2, CA1, HSD11B2, ADRB2, BCHE, CCND1, PPARGC1A, MAOA, GSTM1, CA2, GSTM2, CPT2, ABCG2, CDKN1A, CAV1, CXCL2, EIF6, PTGS1, AKR1C1, and AGTR1) (Figure 5e,f). Intersection analysis of the three machine learning screening results ultimately identified 4 core genes (CA1, CCND1, CXCL2, and EIF6) (Figure 5g). These four core genes showed significant expression changes in colorectal cancer. CA1 (Carbonic Anhydrase 1) was downregulated, indicating altered pH regulation in the tumor microenvironment; CCND1 (G1/S-specific Cyclin-D1), CXCL2 (C-X-C Motif Chemokine 2), and EIF6 (Eukaryotic Translation Initiation Factor 6) were upregulated, participating in cell cycle regulation, immune response, and protein synthesis, respectively. Specifically, CA1 downregulation leads to the acidification of the tumor microenvironment, creating favorable conditions for tumor progression; CCND1 upregulation promotes G1/S phase transition, accelerating tumor cell proliferation; CXCL2 upregulation enhances inflammatory response, remodeling the tumor immune microenvironment; and EIF6 upregulation increases protein synthesis efficiency, supporting rapid tumor cell growth (Figure 2c).

### 2.5. Immune Cell Infiltration and Core Gene Associations

**Figure 6:**
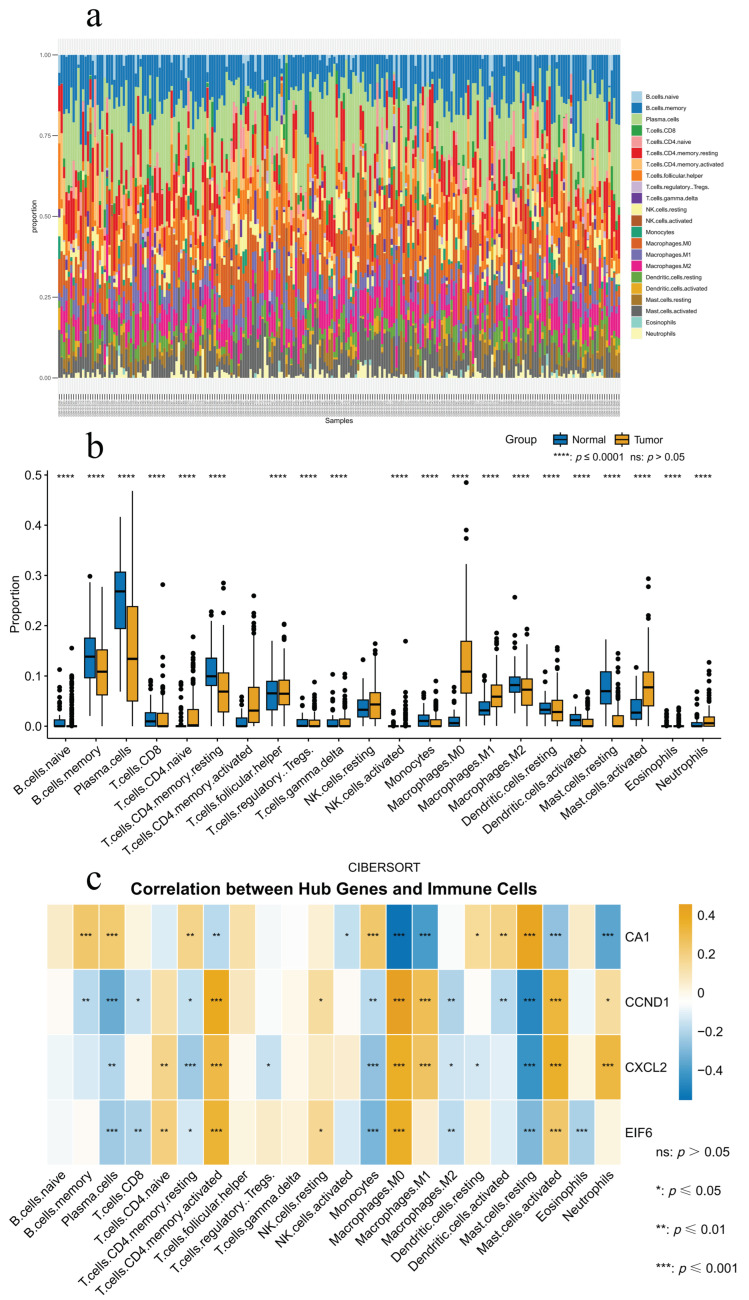
Immune infiltration analysis of hub genes. (**a**) Stacked bar chart illustrating immune cell type infiltration across samples from the GSE41258 dataset. (**b**) Box plot illustrating variations in immune cell infiltration between colorectal cancer and normal samples. (**c**) Heatmap displaying the correlations between hub gene expression and immune cell infiltration.

CIBERSORT analysis identified significant variations in immune cell profiles between colorectal cancer and normal tissues (Figure 6a,b). Tumor tissues showed a significant decrease in adaptive immune cells, including plasma cells and memory B cells, accompanied by an increase in activated CD4+ memory T cells and a reduction in resting CD4+ memory T cells. Among innate immune cells, M0 and M1 macrophages exhibited higher infiltration levels, while mast cells showed polarization, characterized by fewer resting cells and an increase in activated cells.

Correlation analysis (Figure 6c) revealed strong associations between core genes and immune cell infiltration, particularly with M0 macrophages, activated CD4 memory T cells, and monocytes. Additionally, the core genes showed regulatory effects on mast cell polarization. These findings suggest that core genes may reshape the tumor immune microenvironment by modulating immune cell dynamics.

### 2.6. Docking Analysis of Core Target Proteins and Their Ligands

**Figure 7:**
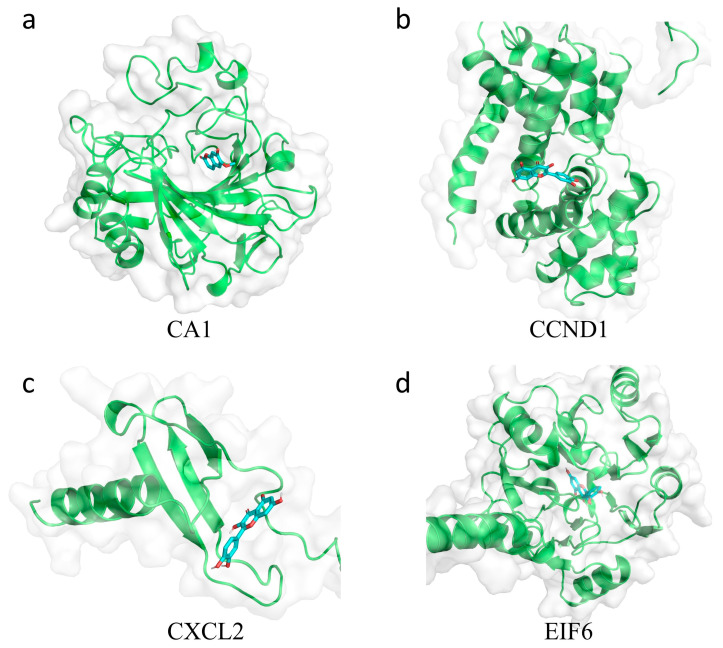
Docking pocket of the target hub proteins. The docking pocket of (**a**) CA1, (**b**) CCND1, (**c**) CXCL2 and (**d**) EIF6. The protein structures are shown in green ribbon representation with transparent molecular surface in white. The ligands are highlighted in cyan and red.

**Figure 8:**
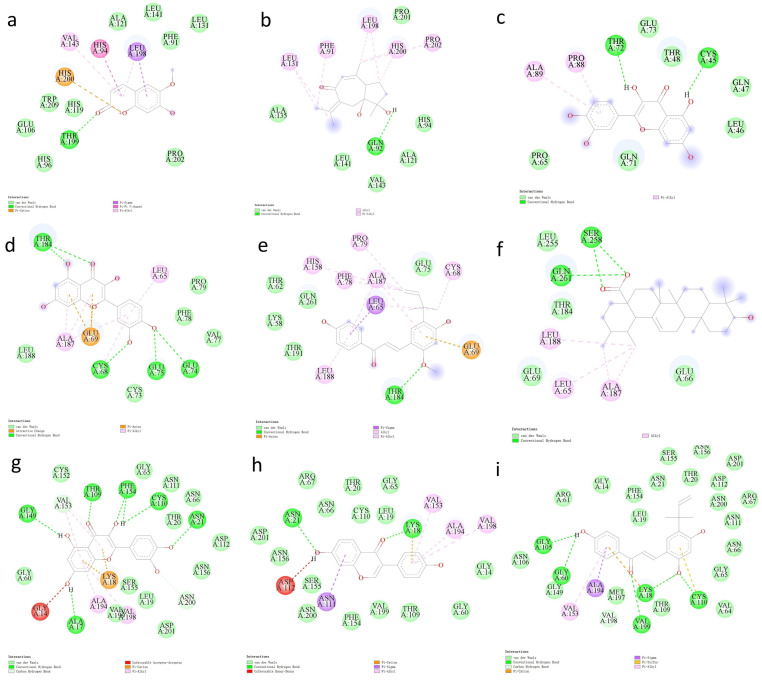
Interaction map of the docking results of the target hub proteins and their corresponding ligand molecules. (**a**) CA1_MOL000040, (**b**) CA1_MOL000960, (**c**) CXCL2_MOL000098, (**d**) CCND1_MOL000098, (**e**) CCND1_MOL000497, (**f**) CCND1_MOL000511, (**g**) EIF6_MOL000098, (**h**) EIF6_MOL000390, (**i**) EIF6_MOL000497.

To investigate interactions between hub genes and their active compounds, we conducted molecular docking. Binding energy thresholds below −5 kcal/mol were used to confirm binding affinity. CA1 and CCND1 structures corresponded to PDB files 1AZM and 2W96, while CXCL2 and EIF6 structures were obtained from the AlphaFold Protein Structure Database. Docking analysis confirmed favorable interactions between all core target proteins and their ligands (Table 1). Figure 7 illustrates the docking pockets of the four proteins, and Figure 8 provides 2D maps of their interactions.

## 3. Discussion

This study thoroughly explored the molecular mechanisms of six medicinal food homologous plants in CRC treatment through integrated network pharmacology and bioinformatics approaches. Through the analysis of active compounds and their targets using databases such as TCMSP and GEO, we identified key therapeutic components and their biological relevance. The pharmacological properties and bioavailability of the active compounds were evaluated (Table 2), revealing that quercetin and licochalcone A meet the standard thresholds for oral bioavailability (OB) and drug-likeness (DL), highlighting their potential for clinical application. Conversely, compounds like procurcumadiol, daidzein, and scopoletin, despite their promising bioactivities, may require structural or formulation modifications to enhance their therapeutic efficacy. These findings underscore the need for further optimization to realize the clinical potential of these compounds in CRC treatment.

Natural products have gained significant research interest due to their potential therapeutic effects and reduced toxicity compared to conventional treatments. Recent studies have documented various medicinal plants with promising anti-tumor activities, particularly in colorectal cancer treatment. Based on current literature evidence, here is a summary of known anti-colorectal cancer plants and compounds.

To contextualize our findings, we have included a summary of key plants and their bioactive compounds with significant therapeutic potential in colorectal cancer (CRC) (Table 3). These plants, such as Curcuma longa (turmeric), Ganoderma lucidum (Reishi mushroom), and Coptis chinensis (Chinese goldthread), exhibit anticancer properties through mechanisms like apoptosis induction, oxidative stress modulation, anti-inflammatory effects, and immune microenvironment regulation. For example, curcumin suppresses NF-κB signaling and induces apoptosis, while sulforaphane from broccoli activates the Nrf2 pathway, enhancing oxidative stress resistance.

The table also highlights the immunomodulatory potential of these compounds. Ganoderma polysaccharides enhance immune cell infiltration, and berberine modulates gut microbiota to suppress CRC progression [47,48]. These findings underscore the value of integrating natural compounds into CRC therapy, both as chemopreventive agents and complementary treatments.

The tumor immune microenvironment plays a pivotal role in colorectal cancer progression, with recent studies highlighting the importance of oxidative stress and inflammation as key factors shaping this environment. Antioxidants have been shown to provide protective effects through various cellular mechanisms [49]. In light of this understanding, our study provides new insights into how medicinal food homologous plants might modulate the immune microenvironment through their antioxidant properties.

CIBERSORT analysis identified notable alterations in immune cell composition between colorectal cancer and normal tissues. In the adaptive immune compartment, plasma cells and memory B cells showed markedly decreased proportions in tumor tissues, suggesting the potential suppression of humoral immunity in the tumor environment. The T cell compartment demonstrated a notable shift, with significantly elevated levels of activated CD4+ memory T cells and reduced levels of resting CD4+ memory T cells in tumor tissues. This pattern may indicate an ongoing adaptive immune response against tumor cells.

Regarding innate immune cells, M0 and M1 macrophages exhibited substantially increased infiltration, while mast cells demonstrated distinct polarization patterns, characterized by decreased resting phenotype and elevated activated phenotype. These changes in macrophage and mast cell populations likely reflect the complex inflammatory state within the tumor microenvironment. These changes in macrophage and mast cell populations likely reflect the complex inflammatory state within the tumor microenvironment. The active compounds from our selected plants may impact these immune cell populations through their antioxidant effects.

This hypothesis is supported by our correlation analysis between core genes (CA1, CCND1, CXCL2, and EIF6) and immune cell infiltration, which demonstrated significant associations with M0 macrophages, activated CD4 memory T cells, and monocytes. These core genes also showed significant differential regulatory effects during mast cell polarization. The convergence of these findings suggests that our identified compounds might exert their anti-tumor effects through dual mechanisms: direct modulation of immune cell populations and regulation of oxidative stress responses. However, the precise molecular pathways linking antioxidant activity to immune modulation require further experimental validation.

Our study successfully integrated network pharmacology with bioinformatics approaches, employing multiple machine learning algorithms for core gene screening while identifying the specific patterns of immune cell modulation. However, this study is limited by its reliance on computational predictions, which need validation through experimental studies. Molecular docking provides initial insights, but molecular dynamics simulations and further exploration of compound interactions are needed to confirm and expand these findings. Despite these constraints, this study provides a robust foundation for advancing the therapeutic potential of natural compounds in CRC treatment.

## 4. Materials and Methods

### 4.1. Acquisition of Active Components and Drug Targets

Active components and their associated targets for six medicinal food homologous plants (*Astragalus membranaceus*, *Ganoderma lucidum*, *Dioscorea opposite*, *Curcuma longa*, *Glycyrrhiza uralensis*, and *Pueraria lobata*) were obtained from the TCMSP database (https://tcmsp-e.com/, accessed on 15 December 2024). UniProt served as the reference database for all acquired target genes normalization [50] (https://www.uniprot.org/, accessed on 15 December 2024) and converted to official gene symbols.

### 4.2. Retrieval of Disease-Related Targets from Public Databases

Colorectal cancer-related target genes were retrieved from the following databases: GeneCards database [51] (https://www.genecards.org/, accessed on 25 November 2024); OMIM database [52] (https://omim.org/, accessed on 25 November 2024); TTD database [53] (https://db.idrblab.net/ttd/, accessed on 25 November 2024), using “colorectal cancer” as the search keyword to integrate disease-related genes.

### 4.3. Differential Gene Analysis

The colorectal cancer gene expression dataset GSE41258 was obtained from the GEO database. Data normalization was carried out with R 4.4.2 [54], followed by differential analysis using the limma package [55]. Differential genes were filtered using criteria of |log2 fold change (FC)| ≥ 0.585 and *p*-value < 0.05 [56,57,58].

### 4.4. Weighted Gene Co-Expression Network Analysis (WGCNA)

Co-expression network analysis was performed on the GSE41258 dataset using the WGCNA R package [59]. A weighted connectivity network was generated by determining the appropriate soft threshold, with gene clusters subsequently identified through dynamic dendrogram analysis. Following statistical correlation studies between Module Eigengenes (MEs) and phenotypic features, modules significantly correlated with colorectal cancer (correlation coefficient > 0.7) were selected.

### 4.5. Construction of Protein–Protein Interaction Network

The intersection of drug targets, disease-related genes, differentially expressed genes, and WGCNA key module genes was calculated to obtain an intersection gene set. The STRING database [60] (https://string-db.org/, accessed on 24 December 2024) was used to construct a protein–protein interaction network with a confidence score threshold ≥ 0.4. Network visualization was performed using Cytoscape 3.10.3 [61].

### 4.6. Functional Enrichment Analysis

GO enrichment analysis [62] and KEGG pathway enrichment analysis [63] of the intersection gene set were conducted with the clusterProfiler package [64] in R. The significance threshold was set at *p*-value < 0.05. A bar plot from GO enrichment analysis and a cnet plot from KEGG pathway enrichment analysis were generated for visualization.

### 4.7. Machine Learning Analysis for Hub Gene Selection

The intersection gene set underwent additional screening through multiple ML methods, including LASSO, SVM-RFE, and RF algorithms. From the intersection targets, we employed these machine learning approaches to identify signature genes differentiating CRC patients from healthy controls. The consistent gene candidates detected across all three algorithms were established as key targets of these six medicinal food homologous plants against CRC. The recursive feature elimination process based on SVM was executed in R 4.4.2, employing e1071 and caret functionalities. The specific steps were as follows: Five-fold cross-validation (k = 5) was performed on the preprocessed expression matrix; feature selection was conducted using the svmRFE function with parameter halve.above = 100; features from 1 to 49 were evaluated using the FeatSweep.wrap function; error rates and accuracy were calculated for each feature set size; optimal feature number was determined based on minimum error rate; corresponding core gene sets were obtained. LASSO regression analysis was conducted using the glmnet package in R, with the optimal lambda value determined via five-fold cross-validation, identifying candidate genes based on non-zero coefficients. Random Forest (RF) analysis, performed with the randomForest package in R, generated models to calculate gene importance scores, selecting those above 0.5 as candidates. The final core gene set was established by intersecting the results of the three machine learning methods. A Venn diagram illustrating the overlap among these methods was generated using the Venn package in R.

### 4.8. Immune Infiltration Analysis

The CIBERSORT algorithm [65] was employed to analyze the infiltration of 22 immune cell types in GSE41258 dataset samples, aiming to explore potential associations between target hub genes and immune microenvironment alterations in CRC patients. First, expression matrices were normalized to eliminate batch effects, and the LM22 signature matrix was used as a reference dataset. Immune cell proportions in each sample were obtained through permutation testing (perm = 1000). Analysis included: comparison of immune cell composition differences between CRC and control groups (*t*-test), generation of immune cell infiltration heatmaps showing overall distribution patterns, and calculation of Spearman correlations between core gene expression levels and immune cell infiltration levels (*p* < 0.05). Statistical analyses and visualizations were implemented in R 4.4.2 with compatible statistical packages.

### 4.9. Molecular Docking

Protein structures were obtained from the PDB database [66] or AlphaFold Protein Structure Database [67], while ligand structures were retrieved from the TCMSP database. Discovery Studio 2019 [68] and PyMOL 2.5.0 [69] were employed for PDB file preprocessing and result visualization. AutoDock Vina 1.1.2 [70] was utilized to conduct molecular docking analysis between selected active components and core gene-encoded proteins.

## 5. Conclusions

This study systematically elucidated the mechanisms of action of six medicinal food homologous plants against colorectal cancer using network pharmacology and bioinformatics approaches. Through the TCMSP database, 303 active components were identified from six medicinal food homologous plants, which can act on 453 drug targets, reflecting the complex network regulatory characteristics of traditional Chinese medicine. The 49 potential therapeutic targets identified through differential gene analysis and WGCNA were predominantly enriched in biological processes such as drug response, alcohol response, and fatty acid metabolism, as well as multiple colorectal cancer-related signaling pathways, highlighting the molecular basis of these herbs’ anti-tumor effects. Cross-validation with three machine learning algorithms—LASSO regression, SVM-RFE, and Random Forest—successfully identified four core genes (CA1, CCND1, CXCL2, and EIF6) as key molecular targets through which medicinal food homologous plants may regulate colorectal cancer development and progression.

CIBERSORT immune infiltration analysis demonstrated significant correlations between core genes and the tumor immune microenvironment, specifically involving M0 macrophage polarization and mast cell activation, indicating that these herbs may exert therapeutic effects by modulating the immune microenvironment. Molecular docking simulations further confirmed strong binding affinities between core target proteins and their active components, offering molecular-level insights into the mechanisms of action.

This study not only clarified the molecular mechanisms of medicinal food homologous plants against colorectal cancer from a systems biology perspective but also provided theoretical support for their rational clinical application.

## Figures and Tables

**Table 1:** The affinity of the target protein and its corresponding ligand.

Receptor	Entry ID	Ligand	Affinity (kcal/mol)
CA1	P00915	MOL000040 (Scopoletol)	−6.6
		MOL000960 (procurcumadiol)	−6.2
CCND1	P24385	MOL000497 (licochalcone a)	−7.6
		MOL000511 (ursolic acid)	−6.5
		MOL000098 (quercetin)	−7.5
CXCL2	P56537	MOL000098 (quercetin)	−5.9
EIF6	P19875	MOL000497 (licochalcone a)	−5.4
		MOL000098 (quercetin)	−8.0
		MOL000390 (daidzein)	−9.1

**Table 2:** OB and DL of Active Compounds.

	OB (%)	DL
MOL000040 (Scopoletol)	27.77	0.08
MOL000960 (procurcumadiol)	69.82	0.13
MOL000497 (licochalcone a)	40.79	0.29
MOL000511 (ursolic acid)	16.77	0.75
MOL000098 (quercetin)	46.43	0.28
MOL000390 (daidzein)	19.44	0.19

**Table 3:** Plants with Important Anti-Colorectal Cancer Actions.

Plant Name	Active Compounds	Key Mechanisms	Reference
Punica granatum (Pomegranate)	Ellagic Acid	Antioxidant, anti-proliferative, induces tumor cell cycle arrest and apoptosis	[36,37]
Scutellaria baicalensis (Chinese Skullcap)	Baicalein	Inhibits tumor cell migration and invasion, suppresses PI3K/Akt signaling pathway	[38,39]
Coptis chinensis (Chinese Goldthread)	Berberine	Anti-inflammatory, modulates gut microbiota, inhibits NF-κB signaling pathway	[40,41]
Brassica oleracea (Broccoli)	Sulforaphane	Antioxidant, activates Nrf2 pathway, enhances resistance to oxidative stress	[42]
Ganoderma lucidum (Reishi Mushroom)	Ganoderma Polysaccharides	Enhances immune function, modulates tumor microenvironment, promotes immune cell infiltration	[43,44]
Taxus chinensis (Chinese Yew)	Paclitaxel	Inhibits mitosis, stabilizes microtubules, induces tumor cell apoptosis	[45]
Panax ginseng (Ginseng)	Ginsenosides	Anti-proliferative, anti-angiogenesis, modulates immune response, suppresses multiple cancer pathways	[46]

## Data Availability

All data are available on request from the authors.

## Supplementary Materials

The supporting information can be downloaded at: https://www.mdpi.com/article/10.3390/ijms26030930/s1.
